# Supplementary material for: Emergence and influence of sequence bias in evolutionarily malleable, mammalian tandem arrays
Source: BMC Biol. 2023 Aug 23;21:179. doi: 10.1186/s12915-023-01673-4 (PMC10463633; doi:10.1186/s12915-023-01673-4)
Supplement: Supplementary file 3 — Additional file 3: Supplemental figures related to main figures 2-7. [file 12915_2023_1673_MOESM3_ESM.zip › AdditionalFile3_supplemental_figures.docx]

Journal: BMC Biology (BMCB-D-23-00360R1)

**Supplementary Materials for**

**Title (BMCB-D-23-00360R1)**

Emergence and influence of sequence bias in evolutionarily malleable, mammalian tandem arrays

**Authors**

Margarita V Brovkina^1,*^, Margaret A. Chapman^2,*^, Matthew L. Holding^3^, and E. Josephine Clowney^4,5, #^

**Affiliations**

1. Graduate Program in Cellular and Molecular Biology, University of Michigan Medical School, Ann Arbor, Michigan, United States of America

2. Neurosciences Graduate Program, University of Michigan Medical School, Ann Arbor, Michigan, United States of America

3. Life Sciences Institute, University of Michigan, Ann Arbor, Michigan, United States of America

4. Department of Molecular, Cellular, and Developmental Biology, University of Michigan, Ann Arbor, Michigan, United States of America

5. Michigan Neuroscience Institute, University of Michigan, Ann Arbor, Michigan, United States of America

*These authors contributed equally to this work.

# To whom correspondence should be addressed:

jclowney@umich.edu

**This file (Additional File 3) includes:**

Figures S2 to S7

**Figure S2:** **Characterization of human genomic isochores.** (A-C) Box and whisker plots describe the relationship between isochore GC% and gene number (A), gene density (B) and isochore length (C). Isochores are binned into deciles according to GC content with decile 10 representing high GC and decile 1 representing high AT. (D,E) UCSC Genome Browser screenshots showing additional example isochores. Gene models are colored according to k-means clusters described below. (D) This isochore is AT-rich and contains a gene bloom; all the genes in this isochore have the same prefix, so prefix diversity (Shannon’s H) is low. (E) This isochore is GC-rich and contains genes from a variety of families, so diversity is high. (F,G) Relationship between isochore length (F) and gene count (G) and gene prefix diversity. Isochores are labeled by most common occurring gene prefix. (H) Full graph in Figure 2E including all gene-containing isochores. Spearmann correlation values per isochore deciles are shown on the right. (I) Residuals (i.e. distance of each point from regression line) of graphs in Figure 2E, S2H. (J) The *IGH* locus, containing V, D, J, and common regions of human IgH. V, D, and J regions are classified as “gene parts” and are not represented in the MANE set; however, the V repeats, where transcription initiates, are in a single AT-rich isochore and lack CpG islands.

**Figure S3: Relationship between GC content of local gene features and the home isochore.** (A-B) Heatmaps displaying GC% over 50bp sliding windows calculated across genes. Graphs in (A) include introns, graphs in (B) exclude introns. Clusters were determined by k-means. Summary line plots depict mean GC% across clusters. <10 genes switched clusters when introns were excluded. (C-F) 2D histograms depicting the relationship between (C) flanking, (D) promoter, (E) coding region, and GC percent of “home” isochore for each gene in the MANE set. (F) Relationship between promoter GC percent and coding sequence GC percent. “Flanking region” spans from 25kb upstream of TSS to 25kb downstream of TES. Correlation coefficient (R^2^) and trend (red line) are shown. (G) Number of unique isochores housing genes in each k-means cluster. Genes in the more extreme k-means clusters are contributed by fewer isochores.

**Figure S4: Data related to Figure 4** (A) Counts of gene number for each paralogous cluster across amniotes analyzed (B) UCSC Genome Browser screenshot showing the hemoglobin β cluster on human chromosome 11. As described previously, the *HBB* cluster is flanked by olfactory receptor genes and these are bracketed by conserved single-copy genes, including *STIM1*, *RRM1* and *FHIP1B* shown here, that are syntenic with the *HBB* cluster since before mammals branched (1). (C) Analysis of gene number and GC% in the HBB-OR cluster across mammals, reptiles, and birds. (D) R^2 values for the relationships between the GC% of each cluster and the GC% of its constituent parts across mammalian species. (E) R^2 values for the relationship between cluster or constituent part GC and the number of genes in the cluster across mammalian species.

**Figure S5: Raw rates of loss of function and missense variation.** Raw gnomAD v2.1.1 counts of missense (A, B) and loss-of-function (C, D) SNVs across MANE genes binned by isochore GC% decile (A, C) or k-means cluster (B, D). (E) Missense and loss of function mutation accumulation in genes of differing degrees of local tandem duplication. (F) Ratio of observed over expected rates of synonymous variants, missense variants, and loss of function variants of genes in k-means cluster 3.3, split by OR and non-OR genes. Observed/expected values are calculated relative to the neutral mutation spectrum by gnomAD. (G) Cumulative frequency of pLI scores for genes binned by their home isochore GC% decile. (H,I) Number of *de novo* point mutations observed per kb in each isochore plotted relative to isochore GC% (H) and isochore GC% binned by decile (I). ~700,000 DNM calls are pooled from all ~11,000 trios sequenced to date (2). (J) Raw density of de novo mutations in isochores of different GC content, disaggregated by original study. Reported genome-wide coverage and total size of each dataset are noted on each panel.

**Figure S6:** (A) Manhattan plots of maternal and paternal standardized relative recombination rates for all chromosomes, as described in Figure 6B. (B-C) UCSC Genome Browser screenshots of (B) OR and MS4A loci, flanked by GC-rich isochores and (C) interspersed GC and AT-rich isochores. (D,E) Counts of (D) isochore decile and (E) k-means cluster distribution of genes with 10% highest rate of maternal and paternal within-gene crossovers. (F,G) Counts of (F) isochore decile and (G) k-means cluster distribution of genes with 10% highest rate of within-gene PRDM9 binding.

**Figure S7:** (A) Total CpG dinucleotide rate across isochores by their GC% content, split by isochores containing protein-coding genes and isochores without protein-coding genes. (B) Violin plots displaying total CpG rate across isochores, binned by decile. (C) Ridgeline plots displaying GTEX tissue expression distribution for genes binned by promoter GC% decile. (D) Distribution of promoter CpG rate of genes binned by k-means cluster assignment, split by degree of tandem duplication. (E) Distribution of promoter CpG rate of genes binned by isochore GC% decile, split by degree of tandem duplication. (F) CpG island density around the TSS (grey line) for genes in each k-means cluster. CpG island density reflects strength of called CpG islands from UCSC unmasked CpG islands (3). Mean CpG rates across gene TSS in each cluster are summarized in line plots, while heatmaps represent island calls around the TSS for each gene. (G-I) Hi-C compartment assignment across 21 tissues (4) for isochores (G), or genes in each isochore (H) or k-means cluster (I). “Always A” and “always B” means the gene was assigned to that compartment in every sampled tissue.

**References**

1. Hardison RC. Evolution of Hemoglobin and Its Genes. Cold Spring Harb Perspect Med. 2012;2(12):a011627.

2. Micklem G, Hillier LW. CpG Islands. Unpublished. http://genomewiki.ucsc.edu/index.php/CpG_Islands (2006).

3. Rodriguez-Galindo M, Casillas S, Weghorn D, Barbadilla A. Germline de novo mutation rates on exons versus introns in humans. Nat Commun. 2020;11(1):3304.

4. Schmitt AD, Hu M, Jung I, Xu Z, Qiu Y, Tan CL, et al. A Compendium of Chromatin Contact Maps Reveals Spatially Active Regions in the Human Genome. Cell Rep. 2016;17(8):2042–59.
