# Supplementary figures and images for: Emergence and influence of sequence bias in evolutionarily malleable, mammalian tandem arrays
Source: BMC Biol. 2023 Aug 23;21:179. doi: 10.1186/s12915-023-01673-4 (PMC10463633; doi:10.1186/s12915-023-01673-4)

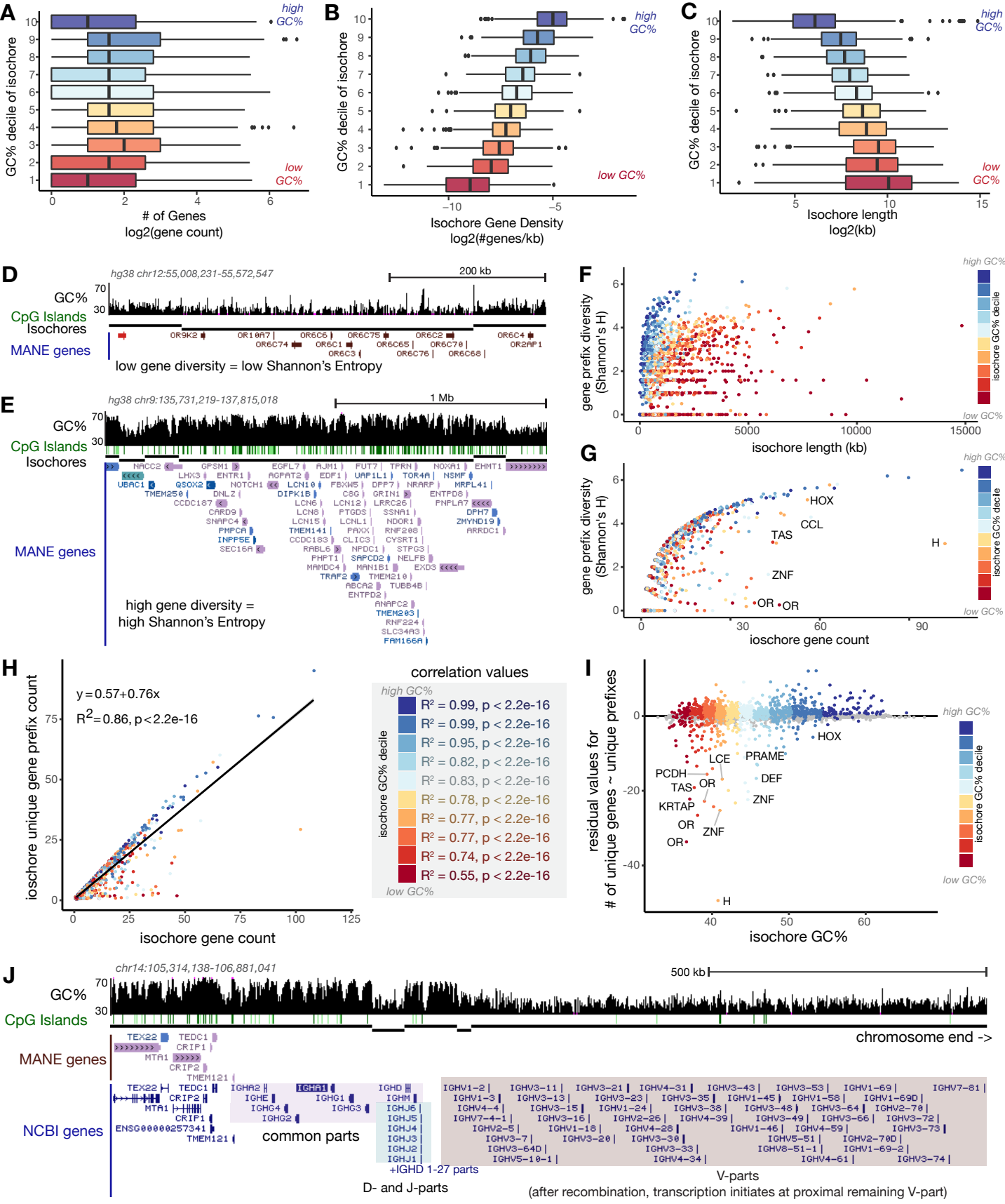

Supplement: Supplementary file 3 — Additional file 3: Supplemental figures related to main figures 2-7. [file 12915_2023_1673_MOESM3_ESM.zip › Figure S2.pdf]

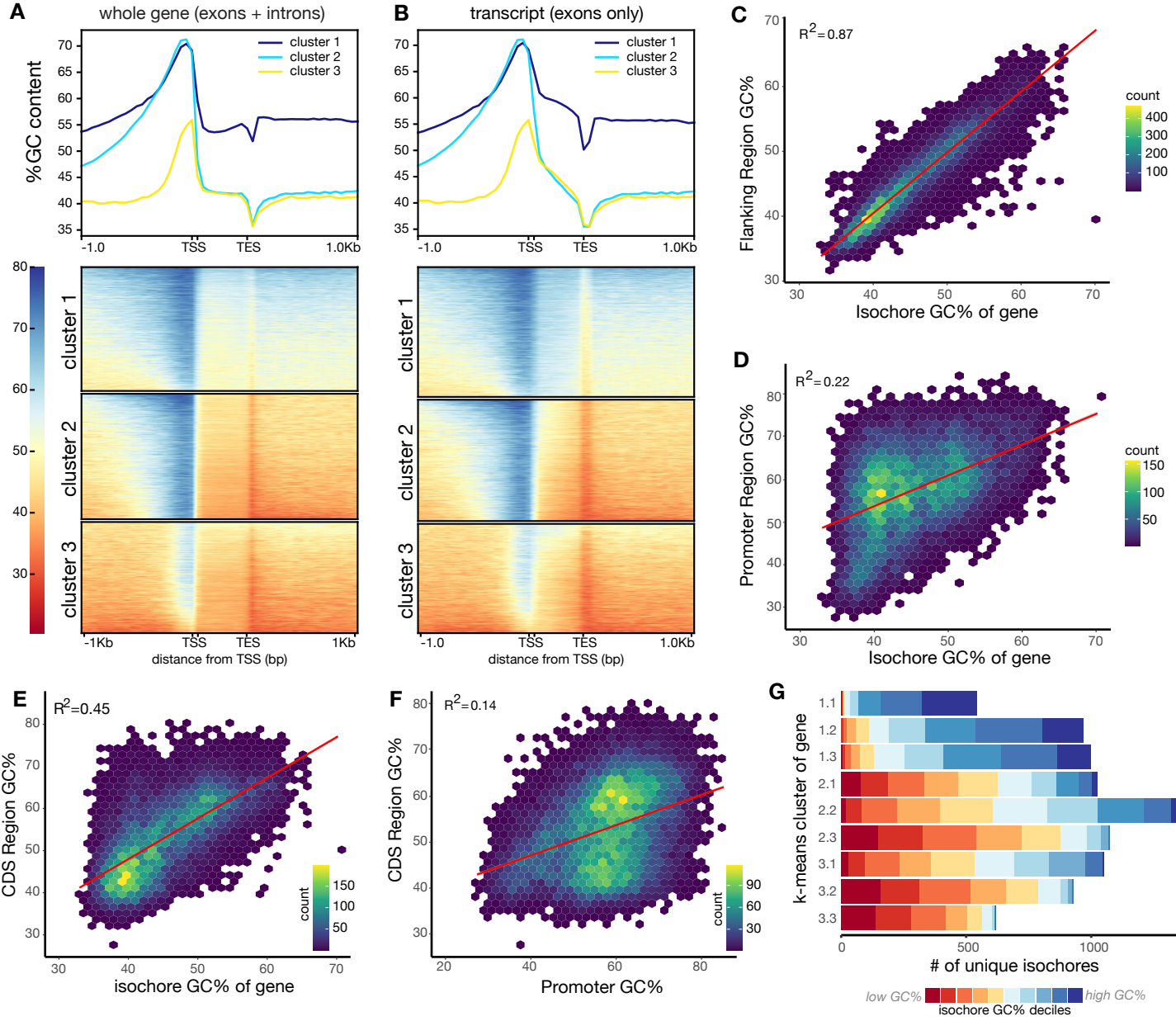

Supplement: Supplementary file 3 — Additional file 3: Supplemental figures related to main figures 2-7. [file 12915_2023_1673_MOESM3_ESM.zip › Figure S3.pdf]

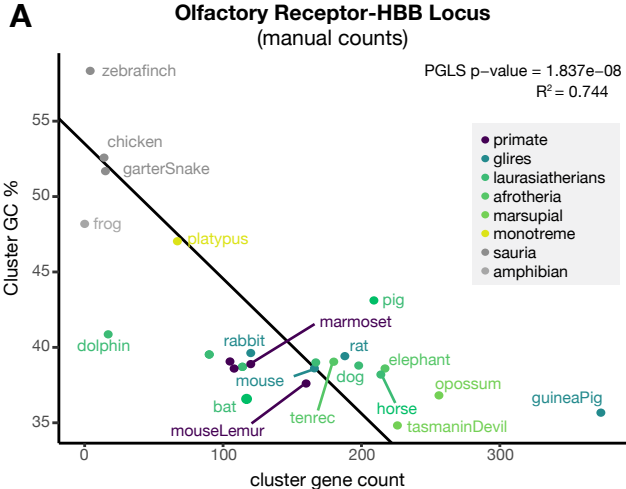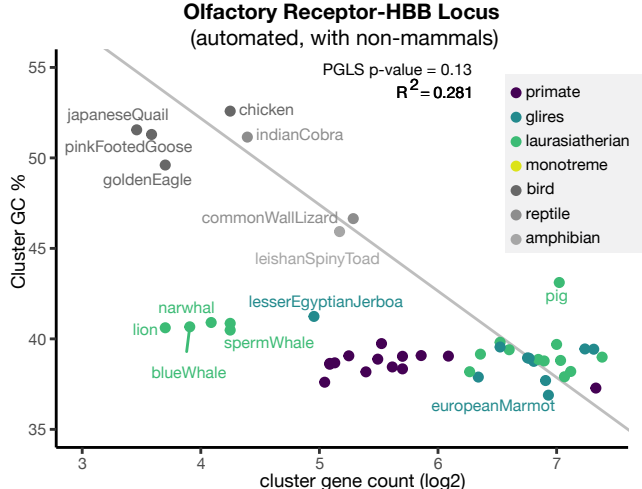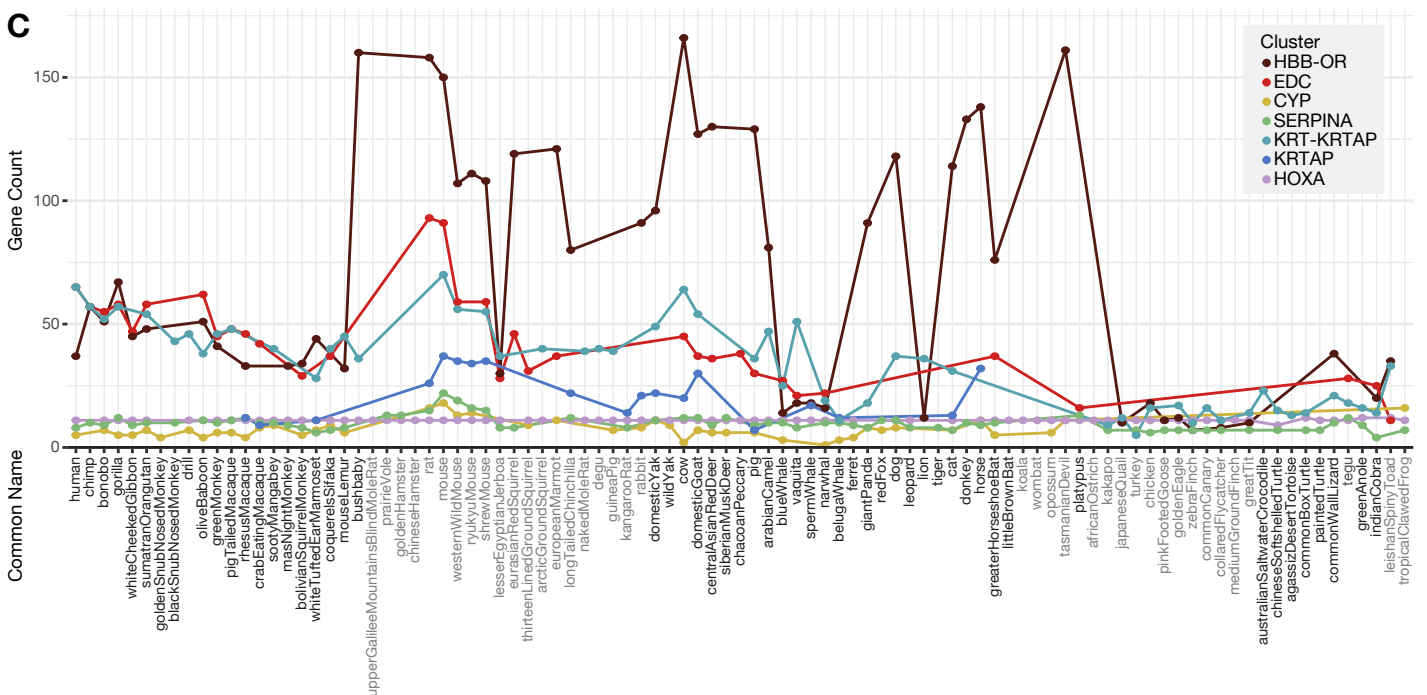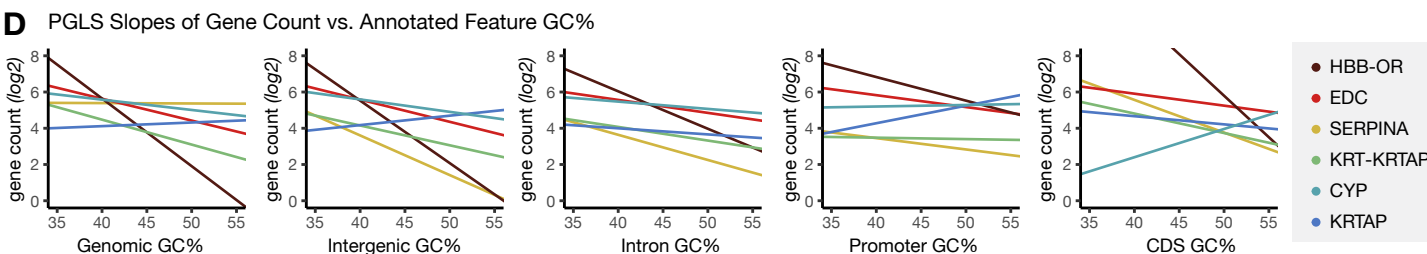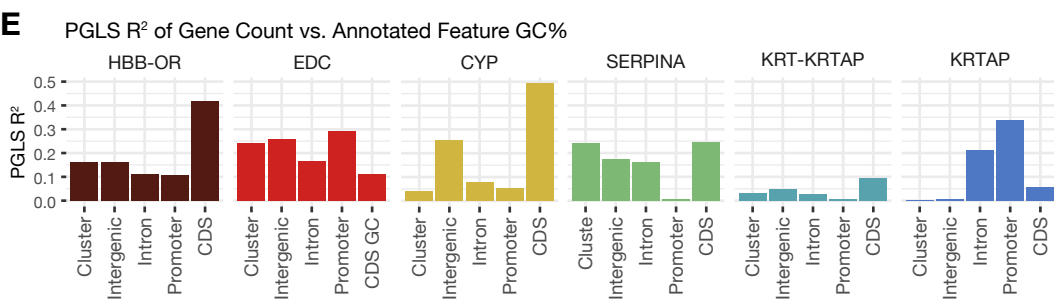

Supplement: Supplementary file 3 — Additional file 3: Supplemental figures related to main figures 2-7. [file 12915_2023_1673_MOESM3_ESM.zip › Figure S4.pdf]

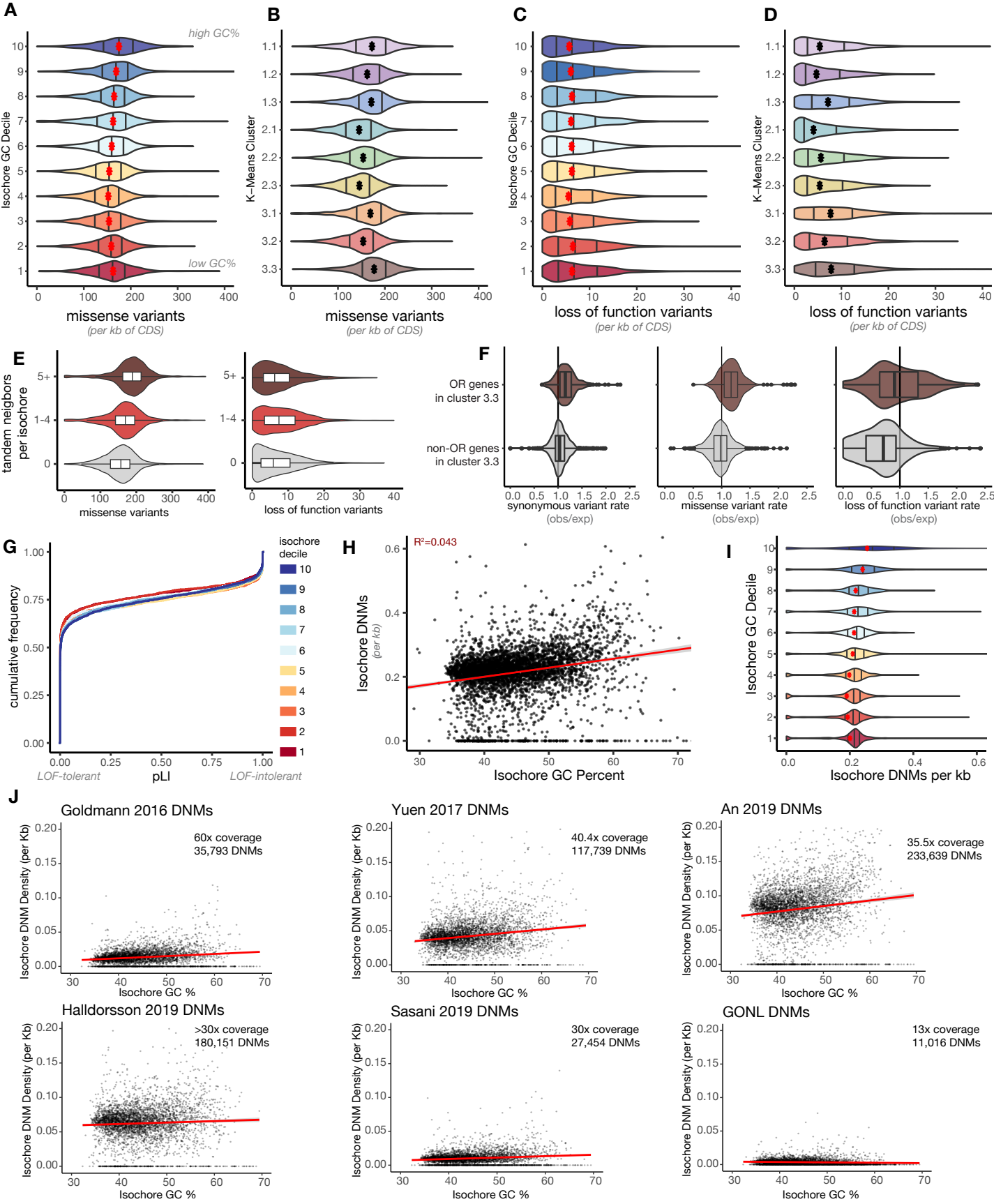

Supplement: Supplementary file 3 — Additional file 3: Supplemental figures related to main figures 2-7. [file 12915_2023_1673_MOESM3_ESM.zip › Figure S5.pdf]

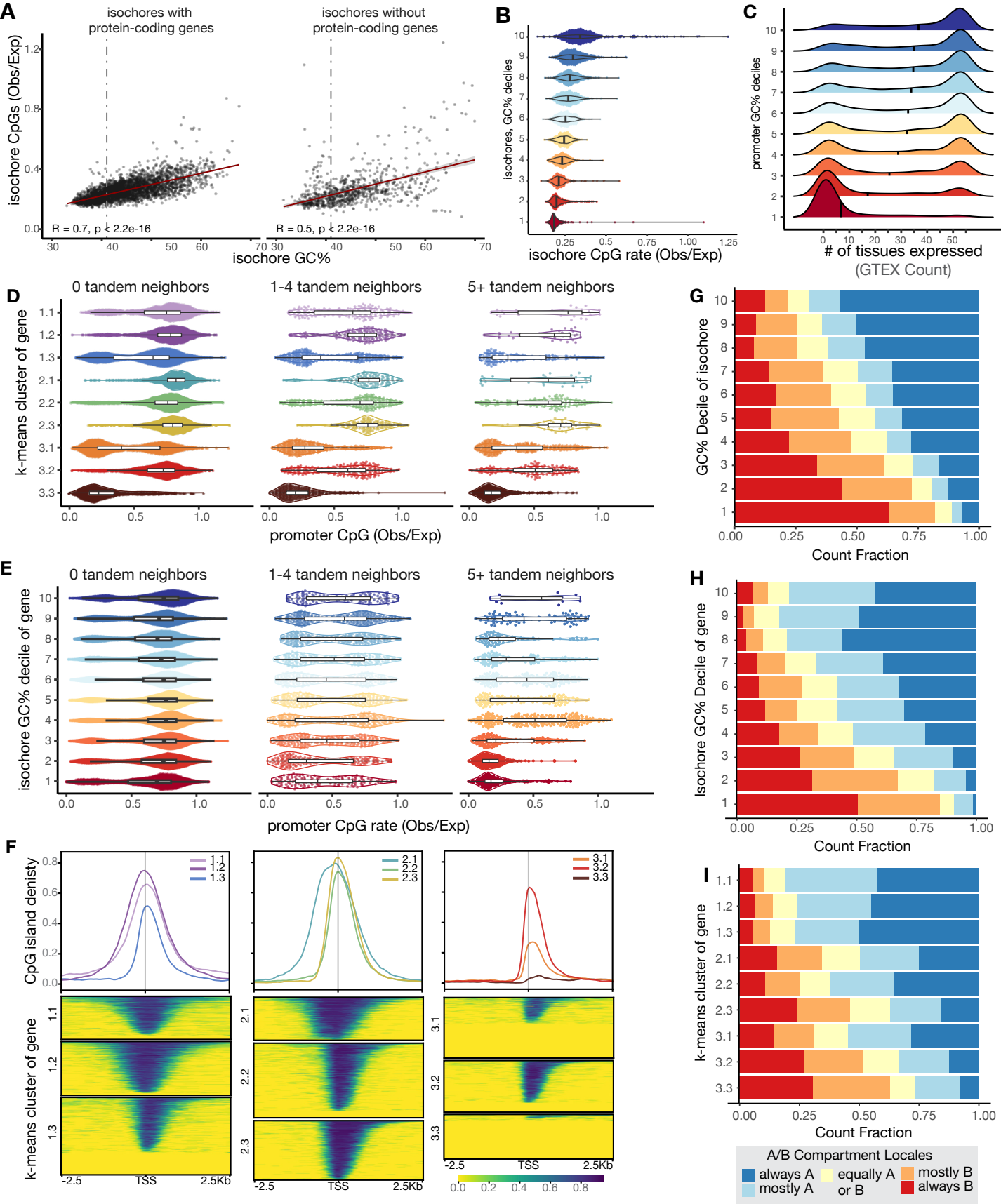

Supplement: Supplementary file 3 — Additional file 3: Supplemental figures related to main figures 2-7. [file 12915_2023_1673_MOESM3_ESM.zip › Figure S7.pdf]
