# Supplementary material for: Emergence and influence of sequence bias in evolutionarily malleable, mammalian tandem arrays
Source: BMC Biol. 2023 Aug 23;21:179. doi: 10.1186/s12915-023-01673-4 (PMC10463633; doi:10.1186/s12915-023-01673-4)
Supplement: Supplementary file 3 — Additional file 3: Supplemental figures related to main figures 2-7. [file Figure S6.pdf › Figure S6.pdf]

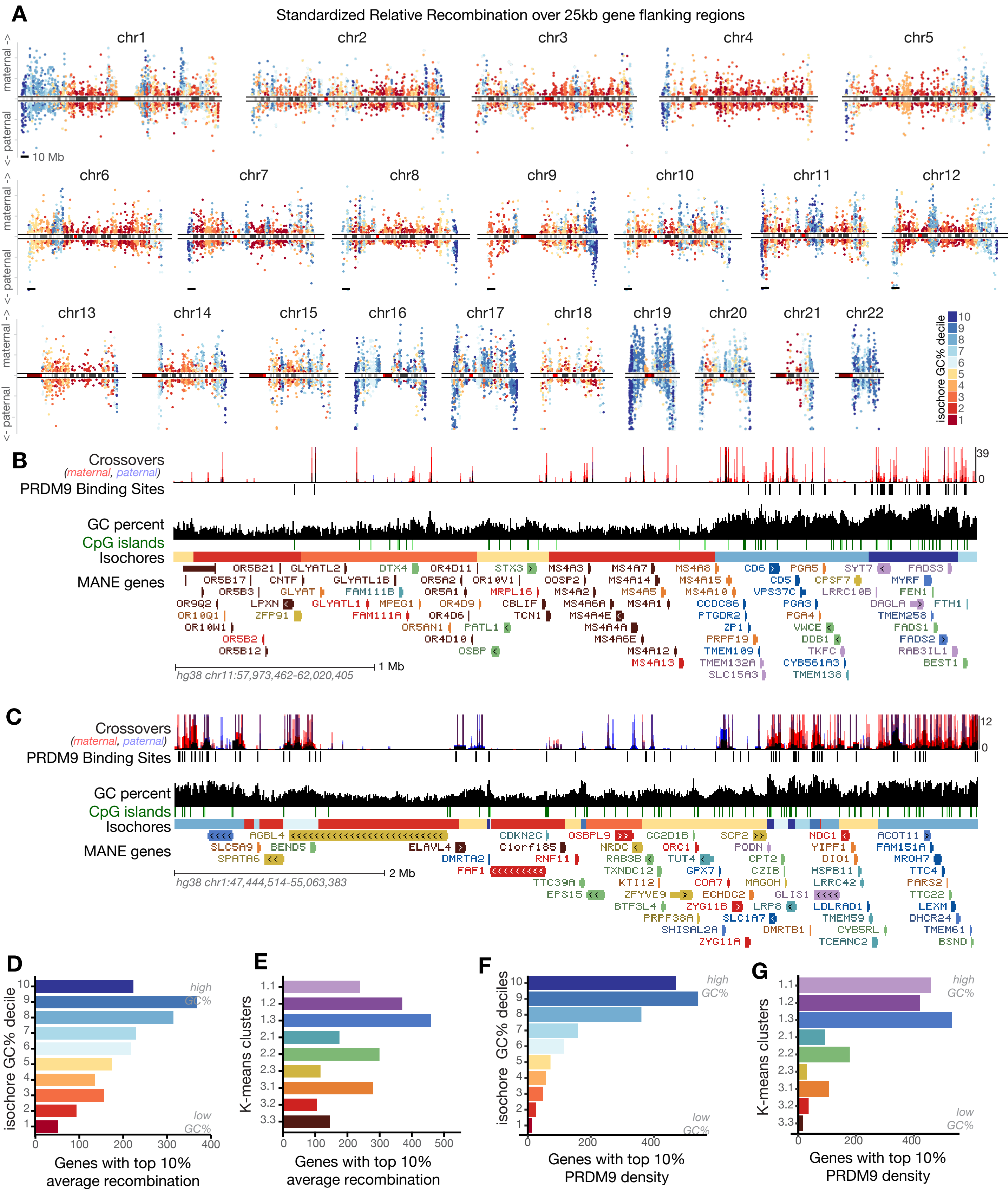

A
Standardized Relative Recombination over 25kb gene flanking regions
chr1
chr2
chr3
chr4
chr5
chr6
chr7
chr8
chr9
chr10
chr11
chr12
chr13
chr14
chr15
chr16
chr17
chr18
chr19
chr20
chr21
chr22
maternal ->
<- paternal
10 Mb
isochore GC% decile
10
9
8
7
6
5
4
3
2
1
B
Crossovers
(maternal, paternal)
39
0
PRDM9 Binding Sites
GC percent
CpG islands
Isochores
MANE genes
OR5B21
GLYATL2
DTX4
OR4D11
STX3
MS4A3
MS4A7
MS4A8
CD6
PGA5
SYT7
FADS3
OR5B17
CNTF
GLYATL1B
OR5A2
OR10V1
OOSP2
MS4A14
MS4A15
CD5
CPSF7
MYRF
OR5B3
GLYAT
FAM111B
OR5A1
MRPL16
MS4A2
MS4A5
MS4A10
VPS37C
LRRC10B
FEN1
OR9Q2
LPXN
GLYATL1
MPEG1
OR4D9
CBLIF
MS4A6A
MS4A1
CCDC86
PGA3
DAGLA
FTH1
OR10Q1
ZFP91
FAM111A
OR4D6
TCN1
MS4A4E
PTGDR2
PGA4
TMEM258
OR10W1
OR5AN1
PATL1
MS4A4A
ZP1
VWCE
FADS1
OR5B2
OR4D10
MS4A6E
PRPF19
DDB1
FADS2
OR5B12
OSBP
MS4A12
TMEM109
TKFC
RAB3IL1
MS4A13
TMEM132A
CYB561A3
BEST1
SLC15A3
TMEM138
1 Mb
hg38 chr11:57,973,462-62,020,405
C
Crossovers
(maternal, paternal)
12
0
PRDM9 Binding Sites
GC percent
CpG islands
Isochores
MANE genes
AGBL4
CDKN2C
OSBPL9
CC2D1B
SCP2
NDC1
ACOT11
SLC5A9
BEND5
ELAVL4
C1orf185
NRDC
ORC1
PODN
YIPF1
FAM151A
SPATA6
DMRTA2
RNF11
RAB3B
TUT4
CPT2
DIO1
MROH7
FAF1
TXNDC12
GPX7
CZIB
HSPB11
TTC4
TTC39A
KTI12
COA7
MAGOH
LRRC42
PARS2
EPS15
ZFYVE9
ECHDC2
GLIS1
TTC22
BTF3L4
ZYG11B
LRP8
LDLRAD1
LEXM
PRPF38A
SLC1A7
TMEM59
DHCR24
SHISAL2A
DMRTB1
CYB5RL
TMEM61
ZYG11A
TCEANC2
BSND
2 Mb
hg38 chr1:47,444,514-55,063,383
D
isochore GC% decile
high GC%
low GC%
Genes with top 10% average recombination
E
K-means clusters
1.1
1.2
1.3
2.1
2.2
2.3
3.1
3.2
3.3
Genes with top 10% average recombination
F
isochore GC% deciles
high GC%
low GC%
Genes with top 10% PRDM9 density
G
K-means clusters
high GC%
low GC%
Genes with top 10% PRDM9 density
